# Liquid Biopsy and Molecular Biomarkers in Mucinous Appendiceal and Colorectal Tumors: Current Evidence and Unmet Challenges in Precision Oncology

**DOI:** 10.3390/cancers18040712

**Published:** 2026-02-23

**Authors:** Diana Maria Orzata, Adrian-Iosif Moldoveanu, Gabriel Veniamin Cozma, Radu Gheorghe Dan, Ovidiu Alexandru Mederle, Laurentiu Vasile Sima

**Affiliations:** 1 Doctoral School, “Victor Babeș” University of Medicine and Pharmacy, 30041 Timisoara, Romania; diana.orzata@umft.ro (D.M.O.); adrian.moldoveanu@umft.ro (A.-I.M.); 2 Department of Surgery I, “Victor Babeș” University of Medicine and Pharmacy, 30041 Timisoara, Romania; gabriel.cozma@umft.ro (G.V.C.); sima.laurentiu@umft.ro (L.V.S.); 3 Center for Hepato-Biliary-Pancreatic Surgery, “Victor Babeș” University of Medicine and Pharmacy, 30041 Timisoara, Romania; 4 Emergency Discipline, Department of Surgery, “Victor Babeș” University of Medicine and Pharmacy, 30041 Timisoara, Romania; mederle.ovidiu@umft.ro; 5 Department of Surgery, Multidisciplinary Centre for Research, Evaluation, Diagnosis and Therapies in Oral Medicine, “Victor Babeș” University of Medicine and Pharmacy, 30041 Timisoara, Romania

**Keywords:** mucinous neoplasms, colorectal cancer, liquid biopsy, circulating tumor DNA, peritoneal metastases, molecular biomarkers, precision oncology

## Abstract

Mucinous appendiceal and colorectal tumors represent a biologically and clinically distinct subset of gastrointestinal malignancies, defined by abundant extracellular mucin, characteristic molecular alterations, and a strong predilection for peritoneal dissemination. Despite these distinctive features, they remain relatively underrepresented in biomarker-driven clinical frameworks, and evidence specific to liquid biopsy performance in this subgroup is fragmented. These features complicate clinical assessment and limit the applicability of tissue-based and circulating biomarkers that perform well in non-mucinous colorectal cancer. Although liquid biopsy is now integral to precision oncology across multiple solid tumors, its role in mucinous disease remains incompletely defined. This review synthesizes evidence on liquid biopsy and molecular biomarkers in mucinous appendiceal and colorectal tumors, with emphasis on circulating tumor DNA (ctDNA) and emerging multi-analyte approaches. Rather than extrapolating from non-mucinous colorectal cancer paradigms, we examine liquid biopsy performance through the lens of mucinous tumor biology and peritoneal compartmentalization. We summarize how peritoneal-dominant spread, limited vascular access, low tumor cellularity, and sequestration of malignant cells within mucin pools constrain biomarker shedding into peripheral blood and contribute to low ctDNA detectability. We discuss the clinical implications for postoperative surveillance after cytoreductive surgery and hyperthermic intraperitoneal chemotherapy, molecular profiling when tissue is limited, and longitudinal monitoring in selected patient subsets. A central focus is the context-dependent interpretation of positive versus negative plasma findings, particularly in low-shedding disease, where false reassurance may occur. We also review strategies beyond plasma ctDNA, including compartment-specific sampling and non-mutational platforms that may improve sensitivity in low-shedding settings. By integrating biological rationale with comparative findings across studies, we identify key evidence gaps and priorities for prospective, biologically stratified validation. We further outline practical considerations for integrating liquid biopsy results into multidisciplinary decision-making in mucinous malignancies. Aligning biomarker class and sampling compartment with mucinous tumor biology may improve interpretability and support more precise clinical management.

## Simple Summary

Liquid biopsy tests tumor-related signals in body fluids, most often blood, to support cancer diagnosis and follow-up. In mucinous and peritoneal-dominant cancers (such as appendiceal tumors and pseudomyxoma peritonei), disease may remain largely confined to the abdominal cavity and release very little tumor material into the bloodstream. Therefore, a negative blood-based liquid biopsy does not necessarily mean that disease is absent. Mucinous appendiceal and colorectal tumors represent a biologically distinct and clinically underrepresented subgroup in which the performance and interpretation of liquid biopsy differ substantially from non-mucinous colorectal cancer, yet disease-specific guidance remains limited. In this review, we explain why detection can be low, summarize which body fluids and test approaches may be more informative in this setting (including blood and peritoneal fluid), and discuss how results should be interpreted for common clinical contexts such as genetic testing when tissue is limited, monitoring after surgery, and assessment of treatment response. Importantly, we propose a clinically oriented, context-dependent interpretation framework that distinguishes between informative positive findings and potentially misleading negative results in low-shedding disease. We also highlight current limitations and research priorities needed to improve clinical use and outline practical considerations for integrating liquid biopsy into multidisciplinary decision-making and patient counseling in mucinous malignancies.

## 1. Introduction

Mucinous appendiceal and colorectal tumors constitute a biologically and clinically distinct subset of gastrointestinal malignancies, defined by abundant extracellular mucin production, characteristic molecular features, and a strong predilection for peritoneal dissemination. In contrast to non-mucinous colorectal cancer, these tumors often follow different trajectories of progression, exhibit variable treatment sensitivity, and carry distinct prognostic implications, limiting the direct transferability of generalized colorectal cancer paradigms to mucinous disease [1,2,3]. Despite these differences, mucinous tumors are frequently discussed within broader colorectal cancer frameworks, and disease-specific guidance for biomarker application remains limited.

Liquid biopsy has reshaped the management of several solid tumors by enabling minimally invasive molecular profiling, treatment monitoring, and detection of minimal residual disease [4,5,6,7]. In colorectal cancer, ctDNA has demonstrated clinical value across disease stages. In mucinous appendiceal and colorectal tumors, however, performance has been less reliable, an inconsistency that appears to reflect fundamental biological constraints rather than technical shortcomings of the assays themselves [5,8,9,10,11]. This divergence highlights the need for a biologically contextualized evaluation of liquid biopsy rather than direct extrapolation from non-mucinous colorectal cancer experience.

Several features of mucinous disease reduce the likelihood that tumor-derived nucleic acids reach the systemic circulation in measurable quantities. Peritoneal-confined growth, sequestration of malignant cells within mucin pools, and comparatively limited vascular invasion can restrict tumor shedding into blood [3,5,9,10,12]. Consequently, plasma-based liquid biopsy frequently exhibits reduced sensitivity in mucinous appendiceal and colorectal tumors, creating interpretive pitfalls and complicating clinical implementation. In this setting, negative plasma results may reflect limited biomarker shedding rather than true biological quiescence, underscoring the importance of cautious interpretation.

These limitations do not negate clinical utility but shift the emphasis toward selective use and biologically informed interpretation. In practice, liquid biopsy may be most useful for molecular profiling when tissue is limited, for risk stratification after cytoreductive surgery and hyperthermic intraperitoneal chemotherapy (CRS–HIPEC), and for longitudinal monitoring in carefully selected patient subsets [7,12,13,14]. A structured, indication-driven approach is therefore required to align assay selection and result interpretation with tumor grade, dissemination pattern, and clinical context.

In this review, we examine the molecular landscape of mucinous appendiceal and colorectal neoplasms, appraise liquid biopsy platforms and their performance limitations, and outline clinically relevant applications and future directions anchored in mucinous tumor biology. Our objective is not only to summarize available data but also to provide a pragmatic interpretive framework to support multidisciplinary decision-making in this distinct group of malignancies.

## 2. Materials and Methods

A focused, clinically oriented literature search was conducted to identify studies evaluating liquid biopsy and molecular biomarkers in mucinous appendiceal and colorectal tumors, including pseudomyxoma peritonei and peritoneal-dominant disease. This review was conceived as a narrative synthesis with a structured search strategy, aimed at contextual interpretation rather than systematic evidence grading. Searches were performed in major biomedical databases, including PubMed/MEDLINE, and complementary indexing platforms (e.g., Google Scholar), covering publications up to December 2025 and limited to English-language articles. Search terms included combinations of: mucinous, appendiceal, colorectal, pseudomyxoma peritonei, peritoneal metastases, liquid biopsy, ctDNA, cell-free DNA (cfDNA), circulating tumor cells, extracellular vesicles, methylation, fragmentomics, minimal residual disease, and CRS–HIPEC. Additional relevant studies were identified through manual screening of reference lists from key reviews and primary articles. We prioritized peer-reviewed clinical and translational studies reporting assay detectability, concordance with tissue, prognostic associations, and/or clinical utility, and interpreted findings in the context of known biological constraints and heterogeneity across mucinous subtypes. Given the rarity of appendiceal mucinous neoplasms and the predominance of retrospective and exploratory cohorts, formal risk-of-bias assessment and quantitative pooling were not considered methodologically appropriate. In light of the additional heterogeneity in study design, cohorts, and assay platforms, findings are presented as a qualitative synthesis rather than a formal meta-analysis. Because the objective was interpretative synthesis and practical clinical framing rather than exhaustive evidence capture or quantitative effect estimation, this review was not designed as a systematic review, and PRISMA reporting was not applied. Accordingly, results are discussed in a biologically stratified manner, emphasizing consistency of patterns across studies rather than numerical aggregation.

## 3. Molecular Landscape of Mucinous Appendiceal and Colorectal Tumors

The molecular landscape of mucinous appendiceal and colorectal tumors differs substantially from that of conventional colorectal adenocarcinoma, not only in the spectrum of recurrent alterations but also in how these changes shape mucin production, growth patterns, and routes of dissemination. Whereas non-mucinous colorectal cancer is often characterized by chromosomal instability and broad genomic complexity, mucinous tumors—particularly of appendiceal origin—tend to exhibit a more constrained set of dominant driver events that define a recognizable molecular and clinical phenotype [2,3,15,16,17]. These molecular distinctions are not merely descriptive but directly influence patterns of tumor shedding and the detectability of circulating biomarkers.

A defining feature of appendiceal mucinous neoplasms is the high prevalence of activating GNAS mutations, most commonly at codon 201. These alterations lead to constitutive activation of cyclic AMP–dependent signaling, promote excessive mucin secretion, and plausibly contribute to the extracellular mucin accumulation characteristic of this disease [16,18]. Across studies, GNAS mutations are enriched in low-grade appendiceal mucinous neoplasms and pseudomyxoma peritonei and remain uncommon in non-mucinous colorectal cancer, supporting their specificity for mucinous tumor biology [16,18]. This distribution is consistent with a role in early tumorigenesis and disease maintenance rather than being restricted to late-stage aggressive progression. From a liquid biopsy perspective, the predominance of a limited set of driver events may facilitate targeted mutation tracking in selected contexts, yet low tumor fraction remains a major limiting factor.

Activating KRAS mutations frequently co-occur with GNAS alterations in appendiceal mucinous tumors and represent another central oncogenic driver [2,19,20,21]. Although KRAS mutations are common across colorectal cancer, their biological consequences in mucinous tumors appear context-dependent, with effects on mucin-associated programs and tumor architecture that are not necessarily associated with rapid invasive behavior [17,19]. Cooperative signaling between KRAS and GNAS has been proposed to explain a characteristic clinical pattern of extensive peritoneal dissemination despite relatively indolent cytologic features and limited systemic spread in many patients [9,22]. This dissemination pattern—dominantly peritoneal rather than hematogenous—provides a biological rationale for reduced plasma ctDNA detectability despite substantial disease burden.

In mucinous colorectal carcinoma, the molecular landscape is typically more heterogeneous than in appendiceal disease. While KRAS mutations remain prevalent, mucinous colorectal tumors more frequently display microsatellite instability (MSI), CpG island methylator phenotype (CIMP), and broader epigenetic dysregulation compared with non-mucinous colorectal cancer [16,23,24]. These features have direct therapeutic implications, including differential sensitivity to fluoropyrimidine-based regimens and responsiveness to immune checkpoint inhibition in MSI-high disease [2,19,23,24,25]. From a biomarker perspective, this heterogeneity further complicates liquid biopsy interpretation, as distinct biological processes may influence tumor DNA release, fragmentation patterns, and detectability across mucinous colorectal cancer subgroups [5,11]. Accordingly, liquid biopsy performance should not be assumed to be uniform across mucinous histologies but interpreted in light of anatomical origin and molecular complexity.

Conversely, alterations that dominate non-mucinous colorectal cancer—such as truncating APC mutations, extensive copy number changes, and high levels of chromosomal instability—are less prominent in mucinous appendiceal tumors [3,10,17]. This relative genomic stability has important implications for liquid biopsy performance, as lower mutational burden and reduced tumor cell turnover are generally associated with lower ctDNA levels [5,12,26]. In addition, abundant acellular mucin can dilute tumor cellularity and reduce the amount of genetic material available for release into blood, contributing to low ctDNA fractions even in patients with substantial disease burden [5,12]. These features collectively position mucinous appendiceal neoplasms among the most biologically challenging contexts for plasma-based mutation detection.

Beyond canonical driver alterations, emerging evidence highlights roles for epigenetic mechanisms, transcriptional programs, and tumor–microenvironment interactions in shaping mucinous tumor behavior. Aberrant methylation, altered mucin gene expression, and immune microenvironment heterogeneity have been linked to progression and therapeutic resistance [27,28,29,30]. These features further motivate complementary biomarker strategies beyond point mutations, particularly in low-shedding tumors where conventional plasma ctDNA approaches underperform. They also provide a mechanistic rationale for exploring methylation- and fragmentation-based assays that may capture tumor-associated signals independent of high mutational burden.

Collectively, these observations support a central premise: mucinous appendiceal and colorectal tumors are not simply histologic variants of colorectal cancer but biologically distinct entities with important implications for biomarker development and clinical management. A mechanistic understanding of oncogenic drivers, mucin biology, genomic stability, and compartmentalized disease spread is essential for interpreting the limitations of current liquid biopsy approaches and for designing strategies tailored to mucinous disease rather than extrapolated from non-mucinous paradigms. This biology-driven framing underpins the subsequent discussion of liquid biopsy technologies and their context-dependent clinical utility.

## 4. Liquid Biopsy Technologies in Mucinous Tumors

Alongside advances in surgical and systemic therapy, liquid biopsy has become an important component of precision oncology by enabling minimally invasive access to tumor-derived material and serial molecular assessment over time. To facilitate clarity and highlight modality-specific considerations, available liquid biopsy approaches in mucinous tumors are discussed below according to analyte type and sampling compartment.

### 4.1. Plasma ctDNA

Among available modalities, ctDNA is the most clinically mature, with validated roles in molecular profiling, minimal residual disease detection, and recurrence monitoring in colorectal cancer [2,3,18]. In mucinous appendiceal and colorectal tumors, however, direct translation of these applications has yielded inconsistent results, reflecting a combination of biological constraints and assay-dependent limitations that are accentuated in this setting [5].

Plasma-based ctDNA assays rely on the release of fragmented tumor DNA into the systemic circulation, a process influenced by tumor cellularity, vascular invasion, and dissemination pattern. Several of these determinants are intrinsically unfavorable in mucinous disease. Abundant extracellular mucin can physically separate malignant cells from the surrounding vasculature, while low neoplastic cell density reduces the absolute amount of DNA available for release [12,20]. These limitations are particularly pronounced in appendiceal neoplasms and pseudomyxoma peritonei, where dissemination is commonly intraperitoneal rather than hematogenous [1,5].

Anatomical compartmentalization further constrains ctDNA detectability. Peritoneal metastases may remain relatively isolated from the systemic circulation, resulting in low plasma ctDNA levels even when overall disease burden is substantial. Multiple studies report markedly reduced ctDNA detection rates in mucinous appendiceal tumors compared with non-mucinous colorectal cancer, including in advanced-stage disease [5,6,31,32]. Clinically, this creates a meaningful false-negative risk and limits the reliability of plasma ctDNA as a universal monitoring tool in this population.

These biological constraints have important implications for clinical interpretation. In non-mucinous colorectal cancer, postoperative ctDNA positivity is strongly prognostic and is increasingly explored to guide adjuvant decision-making [9,33,34]. In mucinous tumors following cytoreductive surgery and hyperthermic intraperitoneal chemotherapy, however, the prognostic value of plasma ctDNA is less certain. In this context, absence of detectable ctDNA may reflect low tumor shedding rather than molecular clearance, increasing the risk of underestimating residual disease if ctDNA is interpreted as a rule-out test [5,11].

### 4.2. Epigenetic and Fragmentomic Approaches

To address these limitations, alternative approaches are being explored to improve sensitivity in low-shedding settings. Epigenetic strategies—particularly DNA methylation-based assays—have attracted interest because methylation signals may be more abundant and detectable at lower tumor fractions than point mutations [28,35]. Fragmentomic approaches that analyze circulating DNA size profiles and fragmentation patterns similarly aim to capture tumor-associated signals beyond specific driver variants and may mitigate some limitations of mutation-only assays [1,24,34].

In parallel, multi-analyte platforms integrating genomic, epigenomic, and proteomic features have been proposed to increase sensitivity by combining orthogonal biological signals, although performance in mucinous appendiceal and colorectal tumors has not yet been systematically evaluated [25,36].

### 4.3. Compartment-Specific Sampling: Peritoneal Fluid

Finally, non-plasma compartments warrant consideration in peritoneal-dominant mucinous disease. When clinically accessible, peritoneal fluid represents a biologically relevant alternative that may better reflect peritoneal tumor burden and molecular composition than peripheral blood. Several studies report higher concentrations of tumor-derived DNA and improved mutation detection in peritoneal fluid compared with plasma in mucinous appendiceal neoplasms [5,22,37]. Although such sampling is more invasive and not suitable for routine outpatient surveillance, it reinforces a central practical principle: liquid biopsy strategies in mucinous tumors should be aligned with disease biology rather than defaulting to plasma-centric paradigms.

Collectively, these observations underscore that liquid biopsy technologies are not interchangeable across tumor subtypes. In mucinous appendiceal and colorectal tumors, intrinsic biological features constrain plasma ctDNA-based assays and motivate a tailored approach incorporating emerging molecular strategies and, where appropriate, alternative sampling compartments [1,5,12].

## 5. Clinical Applications

**Figure 1:**
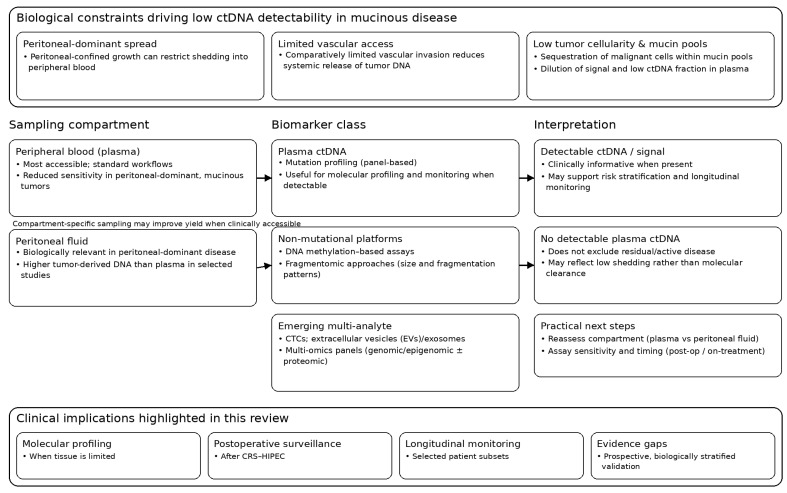
Workflow for liquid biopsy in mucinous and peritoneal-dominant disease, linking key biological constraints to specimen choice (plasma vs. peritoneal fluid), biomarker classes (ctDNA, methylation/fragmentomics, CTCs/EVs), and result interpretation. A detectable signal can be informative, whereas no detectable plasma ctDNA does not exclude disease and should prompt reassessment of compartment, assay sensitivity, and timing. CRS–HIPEC, cytoreductive surgery with hyperthermic intraperitoneal chemotherapy; ctDNA; CTCs, circulating tumor cells; EVs, extracellular vesicles.

The clinical implementation of liquid biopsy in mucinous appendiceal and colorectal tumors should be framed as an indication-driven adjunct rather than a stand-alone decision tool, given biological constraints that distinguish these malignancies from non-mucinous colorectal cancer. Although plasma-based ctDNA assays are routinely used for molecular profiling, minimal residual disease detection, and disease monitoring in several solid tumors, their performance in mucinous disease is often inferior and remains largely investigational in many clinical settings [2,18,38]. From a practical standpoint, liquid biopsy results in mucinous disease should be interpreted within a predefined clinical pathway rather than in isolation. Integration into multidisciplinary tumor board discussions—alongside histologic grade, peritoneal cancer index, completeness of cytoreduction, imaging findings, and treatment timing—is essential to avoid overinterpretation of isolated biomarker signals. Practically, this necessitates asymmetric interpretation: a detectable signal may be clinically informative, whereas a negative result frequently does not exclude clinically relevant disease, particularly in peritoneal-confined presentations (Figure 1).

One commonly proposed application is the postoperative setting following cytoreductive surgery (CRS) and hyperthermic intraperitoneal chemotherapy (HIPEC), where cross-sectional imaging has limited sensitivity for microscopic residual disease. In non-mucinous colorectal cancer, postoperative ctDNA positivity is strongly associated with relapse risk and may precede radiologic progression [9,33,34]. In mucinous appendiceal and colorectal tumors, however, available data are less robust, and studies in appendiceal neoplasms and pseudomyxoma peritonei report markedly lower ctDNA detection rates after CRS–HIPEC, including in some patients who subsequently recur [5,11,37,39]. Accordingly, postoperative ctDNA positivity may reasonably be interpreted as a high-risk signal warranting closer surveillance and multidisciplinary review, whereas ctDNA negativity should not be used to infer molecular remission or justify de-escalation of follow-up in peritoneal-dominant disease.

Importantly, in patient discussions—particularly after CRS–HIPEC—clinicians should clarify that absence of detectable plasma ctDNA does not equate to molecular cure. Transparent communication regarding the biological limitations of plasma-based assays is essential to prevent false reassurance and to support informed shared decision-making.

In the surveillance setting, serial liquid biopsy has been proposed to capture dynamic changes in tumor burden and molecular evolution. While this approach is well supported in conventional colorectal cancer [2,18], evidence in mucinous tumors remains limited and heterogeneous. ctDNA detectability appears enriched in higher-grade disease and/or in the presence of extraperitoneal spread, whereas low-grade, peritoneal-confined tumors may remain ctDNA-negative despite progression [5,6]. From a practical standpoint, plasma ctDNA may therefore function as a marker of aggressive biology rather than as a universal surveillance tool in mucinous malignancies and should be interpreted alongside imaging, histopathology, and clinical course.

In practice, serial testing should complement—rather than replace—standard imaging intervals, and discordance between biomarker findings and clinical assessment should prompt reassessment of sampling compartment, assay sensitivity, and timing.

Liquid biopsy may provide particular value for molecular profiling when tissue acquisition is challenging or when available samples are small, low-cellularity, or potentially non-representative following therapy. In diffuse peritoneal disease, diagnostic biopsies are often limited, and archival tissue may not reflect the contemporary molecular landscape after systemic or regional treatments. In such scenarios, liquid biopsy can support identification of actionable alterations and tracking of clonal evolution [5,12,38]. However, lower mutation detection rates in plasma from mucinous tumors compared with non-mucinous colorectal cancer increase the risk of false-negative findings and incomplete molecular characterization [5,8,9,10]. When clinical decisions depend on molecular results, integration of plasma testing with tissue-based analysis, or alternative compartments when available, remains advisable.

Pre-test counseling in this context should address the possibility of false-negative results and the potential need for confirmatory tissue analysis if therapeutic decisions rely on molecular findings.

Liquid biopsy may also contribute to patient selection and risk stratification when interpreted in conjunction with clinicopathologic variables. Higher-grade mucinous adenocarcinomas and tumors with systemic dissemination show greater ctDNA detectability than low-grade appendiceal neoplasms confined to the peritoneum [5,6,40]. Integrative models incorporating liquid biopsy results with established prognostic factors such as peritoneal cancer index, completeness of cytoreduction, and histologic grade appear more informative than liquid biopsy used in isolation [11,39].

Finally, alternative sampling strategies may add value in selected clinical contexts. Several studies demonstrate higher concentrations of tumor-derived DNA in peritoneal fluid than in plasma in mucinous appendiceal tumors, suggesting that compartment-specific liquid biopsy may better reflect tumor burden and molecular composition in peritoneal-dominant disease [5,22,37]. Although not suitable for routine surveillance, such approaches can be considered when peritoneal fluid access is already clinically indicated, particularly in perioperative or translational settings.

**Figure 2:**
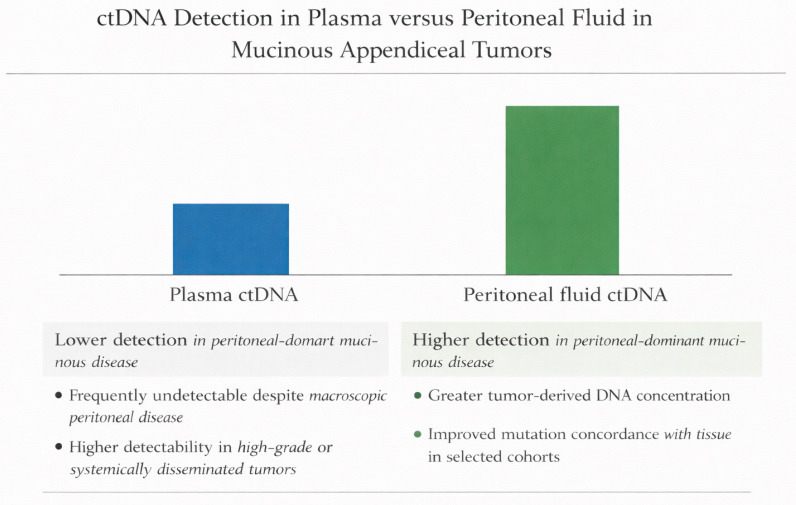
ctDNA detection in plasma versus peritoneal fluid in mucinous appendiceal tumors. Comparative summary of reported detection patterns across representative studies, illustrating reduced plasma ctDNA detectability in peritoneal-dominant mucinous disease and higher tumor-derived DNA concentrations in peritoneal fluid [5,6,11,22,37]. The figure reflects patterns described in the cited literature and does not represent pooled quantitative estimates. ctDNA.

To enhance clarity, Figure 2 summarizes reported ctDNA detection patterns in plasma versus peritoneal fluid across representative studies cited in this review.

Overall, while liquid biopsy is unlikely to replace established diagnostic and surveillance tools in mucinous appendiceal and colorectal tumors in the near term, it can provide incremental value within a multimodal framework when applied selectively, interpreted conservatively, and aligned with mucinous tumor biology rather than extrapolated from non-mucinous colorectal cancer paradigms.

To support consistent application in routine practice, Table 1 summarizes an indication-driven interpretation framework (positive vs. negative results) across the most relevant scenarios.

## 6. Discussion

As outlined in Table 1, negative plasma ctDNA results should be interpreted cautiously in peritoneal-dominant mucinous disease, whereas ctDNA positivity may enrich for aggressive biology.

### 6.1. Biological and Compartmental Determinants of Biomarker Shedding

Liquid biopsy performance depends on the biological context in which tumor-derived material is produced, released, and sampled. In mucinous appendiceal and colorectal tumors, this context differs substantially from non-mucinous colorectal cancer. Extensive extracellular mucin, low neoplastic cellularity, and a strong predilection for peritoneal dissemination shape biomarker shedding and limit the likelihood that tumor-derived DNA reaches peripheral blood [5,6,9,12].

Within this framework, plasma ctDNA assays show consistently reduced sensitivity. Multiple studies report discordance between apparent tumor burden and ctDNA detectability, particularly in appendiceal mucinous neoplasms and pseudomyxoma peritonei [5,6,11,20]. The dominant explanation is compartmentalization rather than assay failure: peritoneal disease constitutes a large reservoir of tumor-derived material that remains relatively isolated from the systemic circulation, limiting DNA release into peripheral blood even in the presence of substantial disease burden [20,37].

Comparative cohorts reinforce this model. When mucinous appendiceal tumors are analyzed alongside non-mucinous colorectal adenocarcinoma, ctDNA detection rates are markedly lower in the mucinous group, including cases with similar radiologic or surgical estimates of burden [5,6,31,32]. Similar observations have been reported in pseudomyxoma peritonei, where plasma ctDNA is frequently undetectable despite macroscopic peritoneal spread [20,37]. Collectively, these findings indicate that ctDNA shedding in mucinous tumors is governed more by anatomical compartment and disease architecture than by tumor volume alone.

Histologic grade and molecular phenotype add clinically relevant heterogeneity. Low-grade appendiceal mucinous neoplasms typically exhibit limited vascular invasion and slow tissue remodeling, constraining apoptotic and necrotic DNA release into circulation [6,41]. In contrast, high-grade mucinous adenocarcinomas, including tumors with signet ring features, show greater genomic instability and more frequent extraperitoneal dissemination; several retrospective series associate these features with higher ctDNA detectability [6,29]. This grade-dependent gradient provides a rationale for stratified interpretation of liquid biopsy results across mucinous subtypes.

Tumor–microenvironment interactions further modulate biomarker shedding. The extracellular mucin matrix creates a diffusion-limited milieu that alters cell–cell interactions, dampens immune surveillance, and reduces apoptotic turnover compared with solid epithelial tumors [12,20]. These properties can lower circulating tumor fractions even when disease is clinically active.

Evidence from non-plasma compartments supports the compartmentalization model. Multiple studies report substantially higher concentrations of tumor-derived DNA in peritoneal fluid than in matched plasma samples from patients with mucinous appendiceal tumors and peritoneal carcinomatosis [5,22,26,42]. In comparative analyses, actionable mutations detected in peritoneal fluid were frequently absent in plasma, highlighting the limitations of systemic sampling in compartmentalized disease [5,26]. These findings are consistent with earlier cytologic and proteomic analyses demonstrating enriched tumor signals in peritoneal effusions relative to peripheral blood [36,43].

Importantly, these constraints are not uniform across all mucinous tumors. Available data support a continuum of shedding influenced by histologic grade, molecular complexity, and dissemination pattern rather than histology alone. High-grade mucinous adenocarcinomas with extraperitoneal involvement may show ctDNA detection rates approaching those observed in non-mucinous colorectal cancer, whereas low-grade appendiceal neoplasms confined to the peritoneal cavity frequently remain ctDNA-negative despite substantial disease burden [5,6,29].

Collectively, these observations provide a unifying biological explanation for the heterogeneous performance of plasma-based liquid biopsy in mucinous appendiceal and colorectal tumors. Low ctDNA detectability is often intrinsic to disease architecture, peritoneal compartmentalization, and microenvironmental constraints on biomarker release rather than reflecting assay failure [5,10,41]. Recognizing these determinants reframes the clinical meaning of a plasma ctDNA result: in mucinous disease, plasma assays function primarily as context-dependent indicators of aggressive biology or systemic dissemination, whereas alternative compartments and analytes may better capture peritoneal-dominant disease [5,6,26]. In practical terms, this reinforces the need for multidisciplinary interpretation and cautions against equating plasma ctDNA negativity with biological quiescence in peritoneal-dominant mucinous disease. This biology-driven reframing distinguishes mucinous appendiceal and colorectal tumors from conventional colorectal cancer paradigms and represents a central contribution of this review, emphasizing that liquid biopsy performance must be interpreted through the lens of compartmentalized disease architecture rather than extrapolated from non-mucinous models.

### 6.2. Heterogeneity Across Mucinous Appendiceal and Colorectal Tumors and Its Impact on Liquid Biopsy Performance

A major driver of variable liquid biopsy performance in mucinous malignancies is the biological heterogeneity encompassed by this category. Although these tumors share mucinous histology, appendiceal and colorectal mucinous neoplasms differ substantially in molecular drivers, histologic grade, dissemination pattern, and clinical behavior—features that directly influence biomarker shedding and detectability [2,3,16].

From anatomical and molecular perspectives, appendiceal mucinous neoplasms are distinct from mucinous colorectal carcinomas. Appendiceal tumors typically exhibit a constrained mutational landscape dominated by KRAS and GNAS, relatively low chromosomal instability, and predominantly peritoneal dissemination [2,4,9,17]. In contrast, mucinous colorectal cancers more frequently display microsatellite instability, CpG island methylator phenotype, and broader epigenetic dysregulation, features that may be accompanied by higher cellular turnover and, in some settings, improved ctDNA detectability [16,23,24,33,38]. This divergence likely contributes to the consistently lower ctDNA detection rates reported in appendiceal cohorts compared with mucinous colorectal cancer cohorts [5,6].

Histologic grade represents a second, clinically relevant axis of heterogeneity. Low-grade appendiceal mucinous neoplasms and pseudomyxoma peritonei are characterized by slow proliferation, preserved architecture, and minimal vascular invasion, each of which can limit tumor-derived DNA release into circulation [5,12,44]. By contrast, high-grade mucinous adenocarcinomas—particularly those with signet ring features—exhibit greater genomic instability, higher proliferative activity, and an increased likelihood of systemic dissemination, aligning with higher plasma ctDNA detection rates observed in retrospective series [6,29]. Across comparative studies, ctDNA positivity is enriched in these higher-grade subgroups, supporting grade-stratified interpretation and study design in mucinous disease [5,6].

Molecular heterogeneity further modulates liquid biopsy performance. Tumors dominated by KRAS/GNAS signaling may retain mucin-rich, low-cellularity architectures that constrain biomarker release, whereas tumors harboring additional alterations affecting DNA repair, epigenetic regulation, or cell-cycle control may generate more readily detectable circulating tumor material [12,17,19]. These observations suggest that liquid biopsy performance reflects not only tumor burden but also qualitative aspects of oncogenic signaling and the microenvironmental context shaping biomarker shedding.

Overall, mucinous tumors should not be treated as a single entity for biomarker development or clinical implementation. Variability in anatomical origin, histologic grade, and molecular architecture translates into meaningful differences in ctDNA detectability and provides a coherent explanation for divergent findings across published studies [5,6,29]. Recognizing this heterogeneity is essential for accurate interpretation and supports stratified, biology-informed approaches rather than uniform application of plasma-based paradigms. Importantly, this heterogeneity-based framework underscores a central theme of this review: liquid biopsy strategies in mucinous tumors must be aligned with anatomical origin and histologic grade to avoid misclassification of low-shedding biology as technical assay failure. At the same time, variability across small retrospective cohorts and assay platforms complicates cross-study comparisons, highlighting the need for prospectively designed, biologically stratified validation studies.

### 6.3. Comparative Performance of Liquid Biopsy Technologies in Mucinous Tumors

The biological constraints and heterogeneity discussed above have direct consequences for the performance of different liquid biopsy technologies in mucinous appendiceal and colorectal tumors. Across studies, assay performance is not uniform and depends strongly on disease biology and the anatomical compartment sampled [5,12]. In practice, each modality interrogates a distinct biological fraction of the tumor ecosystem, explaining divergent results across platforms without implying technical failure.

Plasma-based ctDNA is the most extensively studied approach; however, comparative analyses consistently demonstrate reduced sensitivity in mucinous tumors relative to non-mucinous colorectal cancer. Studies focused on appendiceal neoplasms and pseudomyxoma peritonei report low ctDNA detection rates relative to apparent tumor burden, particularly in low-grade and peritoneal-confined disease [5,6]. In contrast, cohorts enriched for high-grade mucinous adenocarcinoma or tumors with extraperitoneal dissemination show higher detection rates, supporting the concept that plasma ctDNA positivity in mucinous disease preferentially reflects aggressive biology and systemic behavior rather than overall disease volume [5,6,29]. This biology-linked pattern provides important context when comparing plasma ctDNA with alternative analytes.

When contrasted with circulating tumor cells (CTCs), key differences in captured biological information emerge. CTC-based assays provide a cellular readout and may better reflect phenotypic heterogeneity and epithelial–mesenchymal plasticity in selected subsets, including high-grade appendiceal adenocarcinoma [29,41]. However, detection rates remain variable, and technical heterogeneity across platforms limits reproducibility and cross-study comparability, restricting their current role to biologically informative or hypothesis-generating applications rather than standardized clinical use [14].

Extracellular vesicles (EVs) represent a mechanistically distinct alternative. Because vesicles are actively secreted rather than passively released through cell death, EV-associated nucleic acids and proteins may remain detectable even when ctDNA fractions are low, including in mucinous phenotypes characterized by limited turnover and compartmentalized spread [7,14,26,45]. Although mucinous-specific data remain preliminary, these findings highlight the potential of vesicle-based strategies to partially bypass shedding limitations inherent to plasma ctDNA.

Epigenetic and fragmentomic approaches further expand the comparative landscape by targeting signals beyond point mutations. Methylation-based cfDNA assays have demonstrated superior sensitivity compared with mutation-only panels in colorectal cancer screening and MRD-related contexts [12,33]. While mucinous-specific validation is limited, early data suggest that methylation signatures may be less dependent on high tumor fraction and therefore more resilient to the biological constraints common in mucinous disease [12,35,41]. Fragmentomic methods that exploit tumor-associated DNA size and fragmentation patterns similarly aim to extract tumor signals independent of specific driver variants and may provide complementary information when mutation-based ctDNA detection is unreliable [24,34,44].

The most pronounced performance differences emerge when plasma assays are compared with compartment-specific sampling. Multiple studies demonstrate substantially higher concentrations of tumor-derived DNA in peritoneal fluid than in plasma in mucinous appendiceal tumors, along with improved mutation detection and greater concordance with tissue-based profiling [5,22]. In some cohorts, actionable alterations detected in peritoneal fluid were absent from matched plasma samples, underscoring the impact of anatomical compartmentalization on biomarker detectability [6,26]. These observations align with earlier cytologic and proteomic studies indicating that the peritoneal cavity represents an enriched reservoir of tumor-derived material in mucinous malignancies [36,43].

Collectively, comparative analyses indicate that plasma ctDNA captures only a partial—and often biology-biased—representation of mucinous disease, whereas alternative analytes and compartment-specific sampling may better align with dominant dissemination patterns [16,18]. Low concordance between platforms likely reflects differential access to distinct biological fractions rather than lack of robustness [7,14]. Accordingly, assay selection should be guided by tumor grade, molecular phenotype, and dissemination pattern, with different modalities providing complementary information when deployed strategically [12,22,41].

Overall, these data reinforce that liquid biopsy performance in mucinous appendiceal and colorectal tumors is biology-limited and compartment-dependent. Recognizing these constraints is essential to avoid inappropriate extrapolation from non-mucinous colorectal cancer paradigms and sets the stage for interpreting liquid biopsy as a context-dependent biomarker rather than a universal quantitative monitor [5,6]. This integrative comparison across platforms represents a key contribution of the present review, emphasizing that divergent assay performance reflects differential access to tumor biology rather than inherent technical inadequacy. At the same time, interpretation of comparative studies is constrained by small sample sizes, retrospective designs, and heterogeneity in assay methodologies, which limit cross-cohort generalizability. Future investigations should prioritize prospectively designed, biologically stratified cohorts with predefined clinical endpoints and harmonized assay platforms to determine how compartment-specific and multi-analyte approaches can be optimally integrated into routine care.

### 6.4. Clinical Interpretability and Context-Dependent Utility of Liquid Biopsy in Mucinous Tumors

Building on the comparative analyses above, the principal limitation of liquid biopsy in mucinous appendiceal and colorectal tumors is often not analytical feasibility but clinical interpretability. Unlike non-mucinous colorectal cancer, where ctDNA kinetics can function as a relatively quantitative surrogate of tumor burden and treatment response, liquid biopsy signals in mucinous disease are strongly shaped by biological context, anatomical compartmentalization, and histologic subtype [5,6,18]. As a result, identical assay outputs may carry different clinical meanings depending on underlying tumor biology rather than assay performance.

Across studies, detectable plasma ctDNA in mucinous tumors is enriched for biologically aggressive disease. ctDNA positivity has been associated with high-grade histology, signet ring cell components, increased mutational complexity, and dissemination beyond the peritoneal cavity [5,6,40]. In these contexts, a positive liquid biopsy result functions primarily as a qualitative biomarker—signaling unfavorable biology or occult systemic behavior—rather than as a continuous quantitative monitor of disease volume. This contrasts with non-mucinous colorectal cancer, where incremental ctDNA changes more reliably track tumor kinetics and therapeutic response [18,38].

Conversely, absence of detectable plasma ctDNA cannot be interpreted as molecular remission or low-risk disease in mucinous malignancies. In cohorts assessed after cytoreductive surgery and hyperthermic intraperitoneal chemotherapy (CRS–HIPEC), several studies report persistently negative plasma ctDNA in patients who subsequently developed radiologic or surgical recurrence [5,11,22]. In peritoneal-confined mucinous disease, ctDNA negativity therefore more often reflects limited biomarker shedding than true absence of residual disease, creating a substantial risk of underestimating recurrence if interpreted as a rule-out test.

Interpretability also varies across mucinous subtypes. High-grade mucinous adenocarcinomas and tumors with extraperitoneal dissemination show higher ctDNA detectability and, in some studies, greater concordance between plasma and tissue genomic profiles than low-grade appendiceal neoplasms confined to the peritoneum [5,6,41]. Failure to stratify by grade and dissemination pattern likely contributes to inconsistent conclusions across studies and complicates cross-study comparisons.

Beyond plasma ctDNA, context-aware interpretation may be enhanced by selecting analytes and sampling compartments aligned with mucinous disease biology. Peritoneal fluid analyses consistently demonstrate higher concentrations of tumor-derived DNA and improved mutation detection compared with plasma in mucinous appendiceal tumors [5,22]. Extracellular vesicle–derived nucleic acids and proteomic signatures may also capture tumor-associated signals in low-shedding settings, although their clinical interpretability and standardization remain under investigation [7,14,36]. These findings reinforce that interpretability improves when biomarker choice aligns with biological and anatomical context rather than defaulting to plasma-centric paradigms.

Finally, available evidence indicates that the clinical value of liquid biopsy in mucinous tumors is maximized when results are integrated with established clinicopathologic variables rather than interpreted in isolation. Multimodal models combining liquid biopsy with tumor grade, peritoneal cancer index, completeness of cytoreduction, and dissemination pattern provide more informative risk stratification than any single modality alone [11,22,40]. Within this framework, liquid biopsy functions as a contextual modifier of risk, complementing—rather than replacing—histopathology, operative findings, and imaging.

Taken together, current evidence supports a reframing of liquid biopsy utility in mucinous appendiceal and colorectal tumors: from a broadly quantitative monitor toward a context-dependent biomarker whose meaning depends on grade, compartment, and dissemination biology. In this paradigm, ctDNA positivity may carry disproportionate prognostic weight, whereas ctDNA negativity requires conservative interpretation grounded in mucinous tumor biology and anatomical compartmentalization. This reframing of interpretability represents a central contribution of the present review, integrating biological constraints, platform heterogeneity, and clinical context into a unified framework for decision-making. Operationalizing this distinction is essential to avoid misinterpretation and to define clinically meaningful roles for liquid biopsy in mucinous malignancies. However, translation into routine practice remains constrained by limited prospective validation, small cohort sizes, and methodological variability across studies, underscoring the need for harmonized, biology-stratified clinical trials with predefined endpoints before widespread implementation.

A practical summary of evidence and clinical readiness across major use cases is provided in Table 2.

### 6.5. Study Limitations and Methodological Variability

Interpretation of the liquid biopsy literature in mucinous appendiceal and colorectal tumors is constrained by substantial methodological heterogeneity. Most available evidence derives from retrospective analyses, small single-center cohorts, or exploratory translational studies, limiting statistical power, external validity, and generalizability [5,6,11]. These limitations are compounded by the biological diversity of mucinous malignancies, in which histologic grade, dissemination pattern, and anatomical compartment strongly influence biomarker detectability.

A major source of variability relates to assay platforms and analytical sensitivity. Studies differ widely in sequencing depth, target breadth, and bioinformatic pipelines, ranging from focused hotspot panels to broader next-generation sequencing approaches [5,18,41]. This heterogeneity complicates cross-study comparison, as low detection rates may reflect true biological scarcity or limited analytical sensitivity. Importantly, comparative analyses suggest that even high-sensitivity platforms do not fully overcome the biological constraints of low-grade, peritoneal-confined disease, indicating that assay optimization alone is unlikely to close the detectability gap in key mucinous subgroups [5,6].

Sampling timing and clinical context represent additional confounders. Liquid biopsy has been performed at heterogeneous time points, including preoperative assessment, early postoperative intervals, surveillance, and radiologic progression [11,17,34]. In mucinous tumors—where shedding may be intermittent and strongly context-dependent—such variability can materially affect reported detection rates and prognostic associations. Few studies systematically relate sampling schedules to completeness of cytoreduction, peritoneal disease burden, or histologic subtype, limiting interpretability and clinical translation.

Cohort composition further contributes to inconsistent findings. Many studies combine appendiceal and colorectal mucinous tumors or pool low-grade and high-grade histologies despite well-established biological and clinical differences between these entities [2,5,6]. Studies that stratify by grade and dissemination pattern more consistently demonstrate improved liquid biopsy performance in high-grade and/or systemically disseminated disease, suggesting that inadequate stratification can obscure biologically meaningful signals and inflate apparent contradictions across the literature [40,41].

Finally, endpoint definitions and outcome ascertainment vary widely. Studies have used radiologic recurrence, clinical progression, or biomarker detection as endpoints, often without harmonized thresholds or standardized surveillance protocols [11,18]. This lack of alignment limits cross-study comparability, impedes quantification of incremental clinical benefit, and complicates development of evidence-based surveillance or treatment algorithms.

Collectively, these limitations indicate that inconsistent liquid biopsy performance in mucinous tumors reflects not only intrinsic biology but also fragmented study design. Importantly, recognition of these methodological constraints strengthens—rather than weakens—the biological framework outlined in this review by clarifying where true biology ends and study variability begins. Future progress will require harmonized analytical pipelines, biologically stratified cohorts, standardized sampling schedules, and prospective designs aligned with peritoneal-dominant dissemination patterns. Such alignment is essential to determine the incremental clinical value of liquid biopsy beyond established clinicopathologic predictors and to define evidence-based integration pathways in mucinous malignancies.

### 6.6. Translational Outlook and Future Directions in Mucinous Disease

Future clinical integration of liquid biopsy in mucinous appendiceal and colorectal tumors will likely depend on a shift from technology-centered optimization toward biology-aligned strategies. Accumulating evidence indicates that directly replicating the plasma ctDNA performance observed in non-mucinous colorectal cancer is intrinsically limited in mucinous disease, given distinct dissemination patterns and microenvironmental architecture [5,6]. A more productive translational approach is therefore to align biomarker class and sampling compartment with the dominant biology of mucinous tumors.

One practical direction is systematic evaluation of compartment-specific sampling. Although peritoneal fluid-based assays are not suitable for routine outpatient surveillance, they may offer higher biological relevance in perioperative settings, molecular profiling when tissue is limited, and structured translational protocols [5,22]. Comparative studies consistently report higher tumor-derived DNA concentrations and improved mutation detection in peritoneal fluid relative to plasma, particularly in low-grade appendiceal neoplasms with peritoneal confinement [36,41]. Prospective studies are needed to define standardized indications, analytical frameworks, and clinically meaningful endpoints for these approaches.

Parallel progress may arise from multi-analyte and multi-omic strategies designed to improve sensitivity in low-shedding contexts. Integrating methylation signatures, fragmentomic patterns, extracellular vesicle cargo, and proteomic features may partially mitigate limitations imposed by low circulating tumor DNA abundance [5,26,34,35,36]. Rather than replacing ctDNA, these modalities are more likely to complement it by enabling composite biomarker models that better reflect mucinous tumor biology. Early comparative data suggest that such approaches can capture biologically relevant signals even when mutation-based ctDNA remains negative [34,36].

From a clinical perspective, the most plausible future role of liquid biopsy in mucinous disease is context-dependent rather than universal. Key translational priorities include defining settings in which liquid biopsy meaningfully alters management—such as biologically informed risk stratification after CRS–HIPEC, molecular profiling when tissue is limited, and monitoring in higher-grade or systemically disseminated subgroups—while avoiding inappropriate de-escalation based on negative plasma results. Achieving this will require prospective, biologically stratified studies integrating liquid biopsy with clinicopathologic variables and standardized surveillance, with endpoints anchored in clinically actionable decisions rather than detection alone. Ultimately, successful translation in mucinous appendiceal and colorectal tumors will depend not on replicating performance benchmarks established in non-mucinous colorectal cancer, but on redefining liquid biopsy utility through the lens of compartmentalized tumor biology—an approach that frames the central thesis of this review.

## 7. Conclusions

Liquid biopsy represents a promising but currently biology-limited tool in mucinous appendiceal and colorectal tumors. The reduced performance of plasma-based assays in this setting is driven primarily by intrinsic disease features—including peritoneal-dominant dissemination, mucin-rich architecture, low tumor cellularity, and limited vascular access—rather than by technical shortcomings alone. Accordingly, absence of detectable plasma ctDNA should not be interpreted as low disease burden, molecular clearance, or favorable prognosis in mucinous malignancies.

Available evidence supports a selective, context-dependent role for liquid biopsy, best applied as a complementary modality alongside operative findings, imaging, and histopathology rather than as a stand-alone surrogate of disease status. Strategies that align biomarker class and sampling compartment with mucinous tumor biology—including peritoneal fluid analysis when clinically accessible and multi-analyte approaches—offer plausible avenues to improve sensitivity and interpretability but remain dependent on prospective validation.

Future progress will require biologically stratified, disease-specific study designs with harmonized analytical methods and clinically meaningful endpoints. By reframing liquid biopsy through the lens of compartmentalized mucinous tumor biology, this review emphasizes that meaningful clinical integration depends on biology-aligned application rather than direct extrapolation from non-mucinous colorectal cancer paradigms. Within such a framework, liquid biopsy may refine risk stratification and decision-making in selected contexts while avoiding misinterpretation in low-shedding disease.

## Figures and Tables

**Table 1:** Context-dependent interpretation of liquid biopsy results in mucinous appendiceal and colorectal tumors.

Clinical Scenario	If Liquid Biopsy Is Positive (Detected Signal)	If Liquid Biopsy Is Negative (No Detected Signal)
Molecular profiling when tissue is limited, low-cellularity, or potentially non-representative	Consider result as true tumor-derived information supporting molecular stratification (actionability, trial eligibility, resistance mechanisms). Confirm with tissue if feasible and clinically warranted.	Does not exclude actionable alterations. False negatives are common due to low shedding, peritoneal confinement, or low-grade biology. If decisions depend on profiling, pursue alternative material when feasible (fresh tissue or compartment-specific sampling).
Post-CRS–HIPEC assessment (risk signaling rather than rule-out MRD)	Interpret as a high-risk relapse signal. Consider intensified surveillance and multidisciplinary discussion of systemic therapy or clinical trial options in the context of the overall treatment plan.	Does not indicate molecular remission. Surveillance should not be de-escalated; recurrence may occur despite negative ctDNA in peritoneal-dominant mucinous disease.
Surveillance/longitudinal monitoring	New or rising positivity may serve as an early biological warning, particularly in higher-grade or systemically disseminated disease. Trigger confirmatory evaluation (imaging, clinical assessment).	Does not equate to absence of progression, especially in low-grade peritoneal-confined disease. Maintain standard surveillance intervals and multimodal assessment.
Patient selection/risk stratification (integrated with clinicopathologic factors)	Supports classification toward more aggressive biology when interpreted alongside grade, disease burden, and dissemination pattern; may justify closer follow-up or intensified evaluation.	Does not reclassify a patient as low risk on its own. Risk assessment should remain anchored in histologic grade, peritoneal cancer index, completeness of cytoreduction, and imaging findings.
Compartment-specific sampling (peritoneal fluid cfDNA; when clinically accessible)	May better reflect peritoneal tumor burden and molecular composition than plasma; can support molecular characterization when plasma is uninformative.	Does not exclude microscopic disease. Best used opportunistically (e.g., perioperative settings) or in structured translational protocols, not for routine outpatient surveillance.

Note: This table provides an evidence-informed interpretive framework rather than prescriptive clinical guidelines. Interpretation should be integrated with clinicopathologic findings and multidisciplinary judgment. The recommendations summarized here are derived from clinical and translational studies discussed throughout this review [5,6,7,8,9,10,11,12,13,14,22,37,39].

**Table 2:** Evidence summary and clinical readiness of liquid biopsy use cases in mucinous appendiceal and colorectal tumors.

Clinical Use Case	Most Informative Specimen/Analyte in Mucinous Disease	What the Evidence Supports (Qualitative)	Key Limitations/Pitfalls	Clinical Readiness (Today)
Molecular profiling when tissue is limited	Plasma ctDNA (selected contexts); consider alternative compartments when available	Can identify actionable alterations in a subset of patients; yield is higher in biologically aggressive and/or systemically disseminated disease; may complement limited tissue	High false-negative risk in low-grade, peritoneal-confined disease; may provide incomplete molecular characterization	Selective adjunct use (not definitive)
Post-CRS–HIPEC risk signaling/MRD-like assessment	Plasma ctDNA (risk signal when positive)	Postoperative positivity may enrich for higher relapse risk in selected series; may precede clinical recurrence by analogy to non-mucinous CRC	ctDNA negativity does not exclude residual disease; shedding is limited after peritoneal surgery; timing and endpoints heterogeneous	Investigational (avoid de-escalation based on negative results)
Surveillance/longitudinal monitoring	Plasma ctDNA in higher-grade or systemically disseminated disease; multi-analyte approaches may add value	New or rising positivity may serve as an early biological warning in selected subgroups	Persistent negativity common in low-grade, peritoneal-dominant disease; clinical action thresholds not standardized	Selective/investigational, depending on subgroup
Compartment-specific sampling for molecular profiling	Peritoneal fluid cfDNA/ctDNA	Often higher tumor-derived DNA concentration; improved detection and concordance with tissue in peritoneal-dominant disease	Requires access (perioperative or clinically indicated); not suitable for routine outpatient surveillance; standardization limited	High translational value; selective clinical use when accessible
Epigenetic assays (methylation)	Plasma cfDNA methylation	May improve sensitivity in low tumor-fraction settings; less dependent on point mutations	Limited mucinous-specific validation; platform heterogeneity; clinical thresholds evolving	Promising/investigational
Fragmentomic approaches	Plasma cfDNA fragmentation patterns	Potential to detect tumor-associated signals independent of specific driver mutations; may complement mutation-based assays	Sparse mucinous-specific evidence; analytical and interpretive frameworks immature	Investigational
Extracellular vesicles (EVs)	EV-associated nucleic acids and proteins	Active secretion may yield detectable tumor signals in low-shedding phenotypes; biologically complementary to cfDNA	Methodological heterogeneity; limited standardization; unclear clinical decision rules	Investigational
Circulating tumor cells (CTCs)	CTC enumeration and phenotyping	May capture cellular heterogeneity in selected high-grade mucinous subsets	Variable detection rates; platform heterogeneity; reproducibility challenges	Investigational/niche use

Note: Evidence levels are based on qualitative synthesis of available studies; no formal evidence-grading framework was applied. Assessments reflect patterns reported in the cited literature and are derived from representative studies referenced in the main text [5,6,7,8,9,10,11,12,13,14,22,29,37,40,41,42,43,44,45].

## Data Availability

Not applicable. No new data were generated or analyzed in this study.

## Abbreviations

The following abbreviations are used in this manuscript:

AMP	Adenosine monophosphate
APC	Adenomatous polyposis coli
cfDNA	Cell-free DNA
CIMP	CpG island methylator phenotype
CpG	Cytosine–phosphate–guanine
CRC	Colorectal cancer
CRS	Cytoreductive surgery
CRS–HIPEC	Cytoreductive surgery with hyperthermic intraperitoneal chemotherapy
CTC(s)	Circulating tumor cell(s)
ctDNA	Circulating tumor DNA
DNA	Deoxyribonucleic acid
EV(s)	Extracellular vesicle(s)
GNAS	Guanine nucleotide-binding protein G(s) subunit alpha (gene)
HIPEC	Hyperthermic intraperitoneal chemotherapy
KRAS	Kirsten rat sarcoma viral oncogene homolog (gene)
MEDLINE	Medical Literature Analysis and Retrieval System Online
MRD	Minimal residual disease
MSI	Microsatellite instability
PRISMA	Preferred Reporting Items for Systematic Reviews and Meta-Analyses
